# Supplementary material for: Development of an Ergonomic Additively Manufactured Modular Saddle for Rehabilitation Cycling
Source: Materials (Basel). 2025 Nov 19;18(22):5242. doi: 10.3390/ma18225242 (PMC12654643; doi:10.3390/ma18225242)
Supplement: Supplementary file 1 [file materials-18-05242-s001.zip › materials-3933234-supplementary.pdf]

materials

MDPI

Supplementary Materials

Development of an Ergonomic Additively Manufactured Modular Saddle for Rehabilitation Cycling

Alberto Iglesias Calcedo [1,†], Chiara Bregoli [2,†], Valentina Abbate [1], Marta Mondellini [3], Jacopo Fiocchi [2], Gennaro Rollo [4], Cristina De Capitani [1], Marino Lavorgna [1,4], Marco Sacco [3], Andrea Sorrentino [1,4], Ausonio Tuissi [2], Carlo Alberto Biffi [2] and Alfredo Ronca [1,*]

[1] Institute of Polymers, Composites and Biomaterials (IPCB), National Research Council (CNR), Via Gaetano Previati, 1/E, 23900 Lecco, Italy; albertoiglesiascalcedo@cnr.it (A.I.C.); valentina.abbate@cnr.it (V.A.); cristina.decapitani@cnr.it (C.D.C.); marino.lavorgna@cnr.it (M.L.); andrea.sorrentino@cnr.it (A.S.); alfredo.ronca@cnr.it (A.R.)
[2] Institute of Condensed Matter Chemistry and Technologies for Energy (ICMATE), National Research Council (CNR), Via Gaetano Previati, 1/E, 23900 Lecco, Italy; chiara.bregoli@icmate.cnr.it (C.B.); jacopo.fiocchi@cnr.it (J.F.); ausonio.tuissi@cnr.it (A.T.); carloalberto.biffi@cnr.it (C.A.B.)
[3] Institute of Intelligent Industrial Technologies and Systems for Advanced Manufacturing (STIIMA), National research Council (CNR), Lecco, Via Gaetano Previati, 1/E, 23900 Lecco, Italy; marta.mondellini@cnr.it (M.M.); marco.sacco@stiima.cnr.it (M.S.)
[4] Institute of Polymers, Composites and Biomaterials (IPCB), National Research Council (CNR), P.le E Fermi 1, 80055 Portici, Italy; gennaro.rollo@cnr.it (G.R.)
* Correspondence: alfredo.ronca@cnr.it
† These authors contribute equally to the work.

Sensitivity Analysis of the Cost Model

To verify the robustness of the cost model described in Section 3.5, a sensitivity analysis was conducted on the main cost parameters affecting the final unit cost of the polymeric module (G8_0.3 configuration). The nominal value of each parameter was derived from experimental fabrication data and literature benchmarks for additive manufacturing cost modelling [1,2]. Table S1 reports the baseline conditions adopted for the model.

Table S1. Baseline parameters used for cost model calculation of the G8_0.3 polymeric module.

Description	Value
Machine operation cost rate - C_o (€/h)	4.75
Build time - T_b (h)	2.97
Labour time - T_l (h)	1.0
Labor cost rate - C_l (€/h)	33
Material cost rate - C_m (€/kg)	51.5
Material density - ϱ (g/cm^3)	0.65
Part volume - V_{part} (cm^3)	185.47
Material factor - K_s	1
Recycling ratio - K_r	1
Total (€/part)	53.32

The total cost per polymeric part was calculated as the sum of the machine operation cost, the labour cost, and the material cost, excluding depreciation and post-processing overheads. The baseline scenario resulted in a total cost of approximately 53.3 € per part, in good agreement with the estimates presented in the main manuscript

 https://doi.org/10.3390/xxxxx

Table S2. Sensitivity analysis (±20%) of key cost parameters for the G8_0.3 polymeric module.

Scenario	Operation O (€)	Labor L (€)	Material M (€)	Total (€/part)
−20% C_o	11.29	33.00	6.21	50.50
+20% C_o	16.93	33.00	6.21	56.14
−20% T_l	14.11	26.40	6.21	46.72
+20% T_l	14.11	39.60	6.21	59.92
−20% C_m	14.11	33.00	4.97	52.08
+20% C_m	14.11	33.00	7.45	54.56

To evaluate the sensitivity of the model to parameter variations, the operation rate (Co), labour time (Tl), and material cost (Cm) were individually varied by ±20%, keeping the other parameters constant (Table S2). The results showed that the total cost ranged between 46.7 € and 59.9 €, with the largest variation driven by changes in labour time. Material cost and machine rate had a relatively lower impact, confirming that manual handling and finishing remain the dominant cost contributors in single-part additive manufacturing. The sensitivity analysis revealed that the unit cost varies between 46.7 € and 59.9 €, corresponding to approximately ±12% around the baseline value (53.3 €) when the main cost parameters (operator cost, labor time, and material cost) are varied by ±20%. Among these factors, labor time (T_l) was identified as the most influential cost driver, in agreement with trends reported in previous additive manufacturing cost [2]

The moderate variation (±12%) of the total cost within the investigated range supports the robustness and linearity of the adopted cost model. These results are consistent with previous analyses of AM cost sensitivity, where labour utilization and build time were identified as the most influential parameters in determining the economic viability of small-batch or customized production.

References

1. Redaelli, D.F.; Abbate, V.; Storm, F.A.; Ronca, A.; Sorrentino, A.; De Capitani, C.; Biffi, E.; Ambrosio, L.; Colombo, G.; Fraschini, P. 3D Printing Orthopedic Scoliosis Braces: A Test Comparing FDM with Thermoforming. *Int. J. Adv. Manuf. Technol.* **2020**, *111*, 1707–1720. https://doi.org/10.1007/s00170-020-06181-1.
2. Ruffo, M.; Hague, R. Cost Estimation for Rapid Manufacturing—Simultaneous Production of Mixed Components Using Laser Sintering. *Proc. Inst. Mech. Eng. Part B J. Eng. Manuf.* **2007**, *221*, 1585–1591. https://doi.org/10.1243/09544054JEM894.
